# Evaluation of recent New Vaccine Surveillance Network data regarding respiratory syncytial virus hospitalization rates in US preterm infants

**DOI:** 10.1080/21645515.2015.1115936

**Published:** 2016-02-18

**Authors:** John P. DeVincenzo, Christopher S. Ambrose, Doris Makari, Leonard B. Weiner

**Affiliations:** a Children's Foundation Research Institute, Le Bonheur Children's Hospital, Memphis, TN, USA; b Department of Pediatrics and Department of Microbiology, Immunology, and Biochemistry, University of Tennessee School of Medicine, Memphis, TN, USA; c AstraZeneca, Gaithersburg, MD, USA; d State University of New York, Upstate Medical University, Syracuse, NY, USA

**Keywords:** palivizumab, preterm, NVSN, RSV, RSV hospitalization

## Abstract

In July 2014, the Committee on Infectious Diseases (COID) updated their guidance on the use of palivizumab, recommending against use in preterm infants 29 to 35 weeks' gestational age (wGA). A primary data source cited to support this significant change was the low respiratory syncytial virus (RSV) hospitalization rate observed in the subpopulation of preterm (<37 wGA) infants evaluated from 2000 to 2005 through the New Vaccine Surveillance Network (NVSN). Here we critically appraise the preterm infant data from the NVSN in the context of data regarding the use of palivizumab in this same time period. Data from the NVSN, an analysis of Florida Medicaid data, and a national survey of US in-hospital palivizumab administration demonstrated that during 2001 to 2007, palivizumab was administered to 59% to 83% of preterm infants born at <32 wGA and 21% to 27% of all preterm infants (<37 wGA). When the NVSN data regarding incidence of RSV hospitalization in preterm infant subgroups were evaluated as a function of chronologic age, preterm infants <32 wGA showed a paradoxical increase in RSV hospitalization with older age, with the highest risk of RSV hospitalization occurring at 18 to 23 months of age. This pattern is most consistent with a reduction in RSV hospitalizations in <32 wGA infants in the first 12 to 18 months of life due to high palivizumab use at these young ages. The NVSN data were not designed to and cannot accurately describe RSV disease burden in preterm infants given the small size of the analyzed subpopulation and the high use of palivizumab during the study period.

## Introduction

The New Vaccine Surveillance Network (NVSN) was originally founded by the Centers for Disease Control and Prevention (CDC) in 1999 to predict the impact of potential new vaccines. In 2000, the NVSN expanded to conduct active, population-based surveillance of hospitalizations and outpatient visits associated with acute respiratory illness in young children.^1,2^ Respiratory syncytial virus (RSV) has been the focus of several NVSN studies, and the resulting data have been extremely valuable in quantifying the burden of RSV disease within the general population of infants and children. However, the size, scope, and design of the NVSN were not intended to measure disease outcomes within subcategories of high-risk infants.

Palivizumab is a monoclonal antibody approved by the US Food and Drug Administration for the prevention of serious lower respiratory tract disease caused by RSV in high-risk infants and children, including those that are preterm. In July 2014, the American Academy of Pediatrics (AAP) Committee on Infectious Diseases (COID) released their updated guidance for use of palivizumab that recommended against use in preterm infants born at 29 to 35 weeks' gestational age (wGA), and that only preterm infants born <29 wGA without chronic lung disease would now be eligible. A principal data source cited by the COID to support this change was data on the incidence of RSV hospitalizations in preterm infants in an NVSN study published in 2013.^1^ There are limitations in the COID's use of NVSN data to describe a small subset of the population, such as preterm infants <35 or <32 wGA who only comprise 4% and 1.6% of all births, respectively.^3^ Because palivizumab reduces RSV hospitalization in preterm infants,^4,5^ it is also necessary to understand the proportion of infants receiving palivizumab when assessing RSV hospitalization rates in these infants.

The COID first published guidelines for palivizumab in 1998 following approval by the US Food and Drug Administration and updated them in 2000, 2003, 2006, 2009, 2012, and 2014. The NVSN data referenced in the 2014 COID guidance, which demonstrated a low incidence of RSV disease in preterm infants 29 to 35 wGA, involved data collected from 2000 to 2005. During this time period, based on multiple studies showing increased disease risk and severity associated with younger gestational age and younger chronologic age,^6-10^ the COID guidelines recommended use of palivizumab during the RSV season in preterm infants ≤28 wGA and <12 months of age at RSV season start, 29 to 31 wGA and <6 months of age at RSV season start, and 32 to 35 wGA and <6 months of age at RSV season start with additional risk factors.

The purpose of the current analysis was to closely examine the NVSN data cited by COID in the context of data describing the use of palivizumab by subgroup during the study period.

## Methods

The estimated proportion of US preterm infants who received palivizumab during 2000 to 2005 was derived from 3 sources: the observed palivizumab utilization from NVSN studies,^1,11^ an analysis of Florida Medicaid data from 2004 to 2005,^12^ and data for 2006 to 2007 from a national survey of in-hospital palivizumab administration in US preterm infants.^13^ The annual numbers of preterm infants surviving the neonatal period by gestational age subgroup were derived from the CDC Natality Files for 2000 to 2005.^14^

To understand in detail the NVSN data on the observed incidence of RSV hospitalizations in preterm infants, the published NVSN RSV hospitalization rates among preterm infant subgroups as a function of chronologic age (as presented in Supplemental Table 3 of the NVSN^1^ publication) were graphed and compared with age intervals in which palivizumab use was recommended during the study period of 2000 to 2005.

## Results

The NVSN, Florida Medicaid (Table 1), and in-hospital administration (Table 2) data demonstrated that palivizumab was used in 21% to 27% of all preterm infants <37 wGA in 2000 to 2007 and varied considerably by gestational age subgroup, with high use in those <32 wGA and low use in those 32 to 36 wGA, consistent with COID recommendations for those seasons. Specifically for the study period of 2000 to 2005, NVSN data demonstrated that 27% of preterm infants <37 wGA received palivizumab.^1^ This estimate was derived from the proportion of infants who had received palivizumab among those hospitalized from causes other than RSV, a proportion that should be reflective of use in the general population. The percentage of AAP guideline–eligible preterm infants <32 wGA without chronic lung disease in the NVSN network in 2001 to 2007 who received palivizumab was estimated at 59%.^11^ The study did not identify any 32 to 35 wGA infants who were eligible by AAP guidelines in their analysis, likely because they were only able to determine the presence of exposure to tobacco smoke and daycare in medical records but not additional prevalent risk factors such as school-age siblings. However, palivizumab use in infants 32 to 35 wGA overall appeared low, as only 7 infants 32 to 35 wGA and <6 months at RSV season start received palivizumab, compared with a calculated 22 palivizumab recipients <32 wGA without chronic lung disease (CLD) and <6 months at RSV season start.^11^ Similar to the usage pattern observed in the NVSN studies, the Florida Medicaid analysis from 2004 to 2005 demonstrated that palivizumab was administered to 68% of preterm infants <32 wGA and 23% of those 32 to 35 wGA. The national survey of in-hospital palivizumab administration in 2006 to 2007 reported receipt rates of 21.4% among infants <37 wGA: 83.3% among infants ≤30 wGA, 36.2% among infants 31 to 34 wGA, and 3.5% among infants 35 to 36 wGA.^13^

**Table 1.** Palivizumab use in preterm infants by gestational age, 2000–2007.

Gestational Age, wk	Average Annual Number of Preterm Infants in U.S.*	Palivizumab Use in NVSN (2000–2007)	Palivizumab Use in Florida Medicaid (2004–2005)^12^
<32	49,510	59%^†^	68%
32–35	301,302	N/A^‡^	23%
Overall <37	350,812	27%^§^	N/A

AAP, American Academy of Pediatrics; CDC, Centers for Disease Control and Prevention; N/A, not available; NVSN, New Vaccine Surveillance Network; wGA, weeks’ gestational age.

*Average number of preterm infants surviving the neonatal period derived from the 2000–2005 CDC Natality File data.^14^

^†^Estimate derived from 2001–2007 NVSN data for preterm infants <32 wGA, excluding those infants with chronic lung disease. An estimate of utilization in infants <32 wGA including those with chronic lung disease was not provided. Inclusion of infants with chronic lung disease would have resulted in a higher proportion as utilization in infants with chronic lung disease was 90%.^11^

^‡^The study did not identify any 32–35 wGA infants who were eligible by AAP guidelines in their analysis, as they were only able to determine the presence of exposure to tobacco smoke and daycare attendance from the medical record and could not determine the presence of other relevant risk factors such as school-age siblings. The overall proportion of all 32–35 wGA infants receiving palivizumab was not reported. However, palivizumab use in infants 32–35 wGA overall appeared low, as only 7 infants 32–35 wGA and <6 months at RSV season start received palivizumab, compared with a calculated 22 palivizumab recipients <32 wGA and <6 months at RSV season start.^11^

^§^Estimate derived from 2000–2005 NVSN data for preterm infants with RSV-negative hospitalizations.^1^

**Table 2.** In-hospital palivizumab use in preterm infants by gestational age, 2006–2007.

Gestational Age, wk	Average Annual Number of Preterm Infants in U.S.*	National Survey of In-Hospital Palivizumab Use (2006–2007)^†^
<31	38,024	83%
31–34	96,856	36%
35–36	215,932	4%
Overall <37	350,812	21%

*Average number of preterm infants surviving the neonatal period derived from the 2000–2005 Centers for Disease Control and Prevention Natality File data.^14^

^†^Estimates derived from 2006–2007 national survey of in-hospital palivizumab administration.^13^

**Figure 1.**
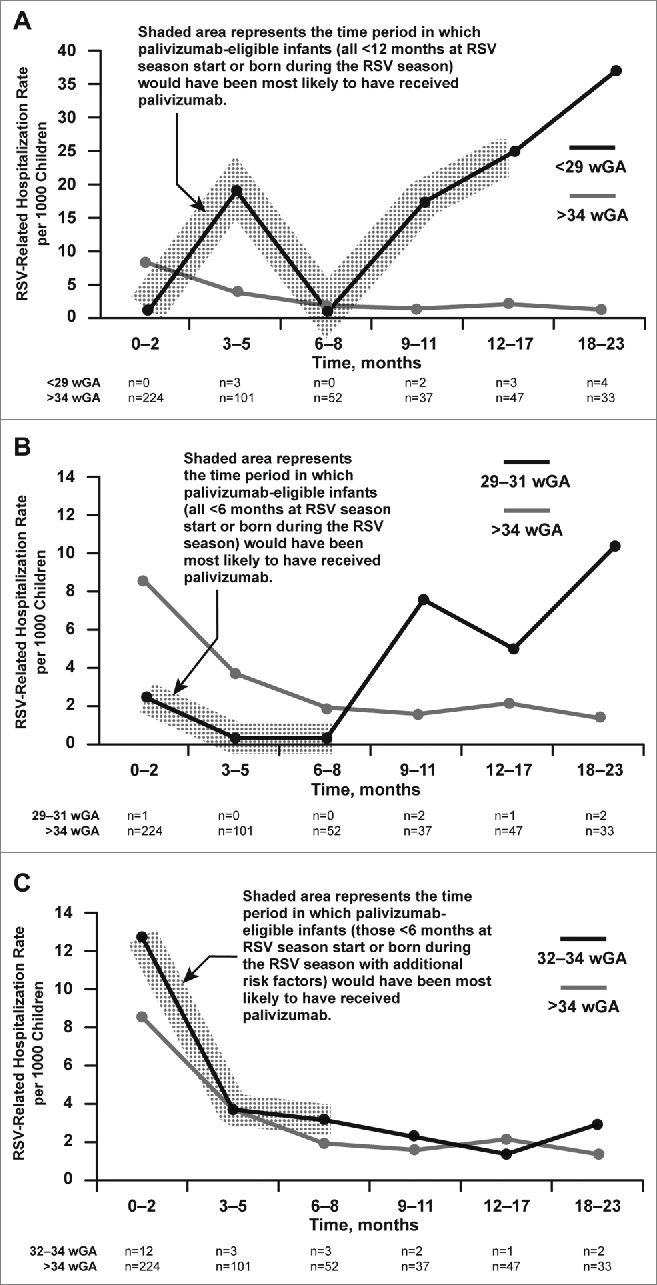
RSV-related hospitalization rate per 1000 children in infants >34 wGA compared with infants A) <29 wGA, B) 29–31 wGA, and C) 32–34 wGA. The number of RSV hospitalizations identified for each time period is displayed below each figure; the NVSN authors did not report the denominator or the 95% confidence intervals of the rates. RSV, respiratory syncytial virus; wGA, weeks' gestational age.

The subgroup analysis of the NVSN data cited by the COID on rates of RSV hospitalization for preterm infants <29, 29 to 31, 32 to 34, and >34 wGA as a function of chronologic age from birth to 23 months of age are included in Figure 1. In infants >34 wGA, in whom there was very little palivizumab use, RSV hospitalization rates as a function of age followed the expected pattern, with the highest rates observed at 0 to 2 months of age. Similarly, for infants 32 to 34 wGA (23 total RSV hospitalizations), in whom palivizumab use was relatively low, the highest RSV hospitalization rate occurred at 0 to 2 months (12.8 per 1000 children), mirroring the age trend observed in infants >34 wGA. However, for infants <29 wGA (12 total RSV hospitalizations) and 29 to 31 wGA (6 total RSV hospitalizations), in whom palivizumab use was high in the first 12 to 18 months of life, RSV hospitalization rates were lower at younger ages and highest at 18 to 23 months, after palivizumab administration would have been discontinued.

## Discussion

The 2014 COID Technical Report states that “data from the NVSN study revealed that for all preterm infants (<37 wGA), the RSV hospitalization rate was 4.6 per 1000 children, which was not significantly different from the hospitalization rate for term infants, which was 5.3 per 1000 children.”^15^ This statement is at odds with data from multiple previous studies showing the preterm infant population to be at higher risk for severe RSV disease compared with full-term infants.^16-37^ Most recently, a 2-season US study showed that preterm infants aged 32 to 35 wGA not receiving RSV prophylaxis had RSV hospitalization rates that were approximately 3 times those reported in the general infant population.^38^ However, the NVSN finding is logical if one considers that the study was conducted when a high proportion of preterm infants, particularly those born at <32 wGA, were receiving RSV immunoprophylaxis with palivizumab at young ages. In addition to the 2 data sources reviewed in the current analysis, similar high rates of RSV prophylaxis receipt of 67% and 63% were demonstrated in <32 wGA infants in Kaiser Permanente Northern California and Tenncare, respectively, between 1996 and 2008.^39^ Additionally, among eligible infants, evidence suggests that palivizumab adherence is highest among infants who are at the greatest risk of severe RSV disease.^39^ The likely effect of palivizumab use on preterm infant RSV hospitalization rates in the NSVN data^1^ is also supported by the observation of higher RSV hospitalization rates at 18 to 23 months of age in <29 wGA and 29 to 31 wGA infants, ages after which palivizumab injections would have been discontinued. Multiple previous studies as well as NVSN data^1^ have demonstrated that in the absence of RSV prophylaxis, younger age is associated with an increased risk of RSV hospitalization in preterm infants.^1-5,11,14-30,33,34,40-47^

A recent *Pediatrics* letter to the editor^48^ raised questions about the COID's interpretation of the NVSN data,^1^ stating “From a scientific standpoint, the authors have misinterpreted the significance of recent data. They cite a recent New Vaccine Surveillance Network (NVSN) study as evidence that the rates of RSV hospitalization are declining. The NVSN study was extremely valuable for its intended purpose of understanding the existing disease burden of RSV in the general population. However, it was performed during a period when palivizumab was being used to prevent RSV hospitalization in high-risk children. This is the most logical explanation for the proportion of high-risk children requiring hospitalization being lower than in studies conducted in the era prior to RSV prophylaxis.” To establish the true burden of RSV disease in preterm infants, one should ideally study a population of preterm infants that did not receive palivizumab. This was done in the recent 2-season study of preterm infants 32 to 35 wGA conducted in 188 US clinics from 2009 to 2011. That study demonstrated a 4.9 per 100 infant-season rate of laboratory-confirmed RSV-related hospitalization in this population, consistent with previous studies and approximately 3-fold higher than the rate observed in US full-term infants.^38^ Available evidence suggests that, among those born <32 wGA, the risk of RSV hospitalization would be higher than that observed among 32 to 35 wGA infants.^17,20,23^

In the current analyses of the NVSN data, unlike <29 and 29 to 31 wGA infants, 32 to 34 wGA infants show the highest rates of RSV hospitalizations in the first several months of life followed by an age-related decline, similar to the pattern observed for infants >34 wGA.^1^ This observation is consistent with previous studies evaluating disease burden in the absence of RSV prophylaxis, as well as the observation that most infants born at 32 to 34 wGA were not receiving palivizumab because of the recommendations that RSV prophylaxis only be provided to those with specific risk factors. Given that the 32 to 34 wGA infants at highest risk were receiving RSV prophylaxis, the overall disease burden observed in the NVSN is presumably lower than would have been observed without palivizumab use; however, as the proportion of 32 to 34 wGA infants receiving palivizumab was low, it is logical that the overall pattern of disease incidence by age group in this population was not substantially altered.

In summary, the NVSN has been quite valuable in evaluating the burden of RSV and other infectious diseases within the overall pediatric population, but NVSN data have major limitations when analyzed by small subpopulations such as preterm infants. Furthermore, the NVSN data evaluated RSV disease risk while the preterm infant population was receiving prophylaxis with palivizumab as standard of care. RSV hospitalization rates among preterm infants within the NVSN dataset appear to reflect the use of palivizumab by gestational and chronologic age, showing lower hospitalization rates when palivizumab use was high and higher rates when palivizumab use was low. As a result, the NVSN data cannot be used to describe the burden of RSV disease in US preterm infants in the absence of data regarding RSV immunoprophylaxis.
